# Extract of a Letter from Dr. De Carro, of Vienna, to Dr. Marcet, of London

**Published:** 1803-09-01

**Authors:** 


EXTRACT OF A LETTER FROM DR. DE CARRO, OF VIENNA, TO DR. MARCET, OF LONDON.

"THE comparative statement of the Jennerian Society, drawn up by Mr. Addington, has already fallen into my hands.

hands. Count Harrach, of this city, (who is passionately fond of the science of physic, to the study of which he has devoted himself for these twelve years, and who has just gone through his examination for the degree of Doctor), has caused this statement to be translated, and distributed gratis. The regency has undertaken to get it distributed in all the parishes, and to have it posted up on the gates of churches, and other public places. All the children of soldiers are now subjected to vaccination. What a difference between this and the time when I was compelled to appear before the police; when vaccination was prohibited within the walls of Vienna, and confounded with the inoculation for the small pox; when I was prohibited giving public notice of my intention of vaccinating the poor gratis; when they had nearly turned out of the city a lady come from Hungary to have her three children vaccinated; when I was prohibited publishing, in the Gazette, the satisfactory result of twenty-one variolic inoculations, tried after vaccination; when the Court, the Ministry, the Police, all conspired against vaccination, whilst the Faculty was doing nothing for its defence! It is under such auspices that I have at last succeeded in introducing the vaccine into this capital, and from hence into all the provinces. At Vienna, we no longer hear of the small-pox. For these two years and a half, I have not met with a single instance of it, and many other physicians will say the same. Pray inform Dr. Jenner, that one of the first occulists of Vienna, Dr. Beer, has assured me, that for these two years, he has not met with a single case of the variolic ophthalmia, whilst he used to reckon annually, at least fifty such cases in his practice! And the doctor's authority is so much the more to be trusted, as he is by no means the gainer in this disappearance of his ophthalmic nosology.

"It gives me sincere pleasure to have afforded some satisfaction to Dr. Jenner, by communicating to him the experiments of Dr. Sacco. Indeed, I had presumed that this account would be particularly agreeable to him; and for that reason, I have transmitted to him further details which I have since received from Sacco. With regard to the nosological innovation proposed, I have already taken some steps to guard vaccinators against the danger of introducing a nomenclature, in which the word *equus* should be used as radical. I have developed the inutility and danger of such an innovation in some Journals, and I have lately sent a letter to the *Bibliotheque Britannique*, which contains all that can be said upon that subject. My chief objection to this change of name, is the confusion which could not fail to arise from it in the minds of uninformed people, who could not understand why a new name should be given to a thing in which they could not perceive the least difference; and this might also have the inconvenience of reviving ideas on the morbid origin of the preservative, which have long been done away. Besides, as the quantity of vaccine matter, now spread over the surface of the globe, is such that it would be impracticable to substitute every where the *virus equinus*, even if there was any advantage in doing it, there would be a sort of competition between *equination* and vaccination. To which of the two would uninformed people give the preference? What an embarrassing dilemma! If there be no real difference between the two methods, why should the names be changed? And the vaccinators themselves, what nomenclature would they adopt, since they never can be able to determine the origin of the matter usesd, unless they have taken it themselves from the heel of the horse or the teat of the cow?—In one word, the thing is more important than it may at first appear, and I am particularly flattered, once more, to see my opinion coincide with that of Dr. Jenner."

*Vienna, June* 18, 1803.

